# Flexibility and Resilience in Corporate Decision Making: A New Sustainability-Based Risk Management System in Uncertain Times

**DOI:** 10.1007/s40171-021-00277-7

**Published:** 2021-08-03

**Authors:** Davide Settembre-Blundo, Rocío González-Sánchez, Sonia Medina-Salgado, Fernando E. García-Muiña

**Affiliations:** 1 Department of Business Administration (ADO), Applied Economics II and Fundaments of Economic Analysis, Rey Juan-Carlos University, 28032 Madrid, Spain; 2 Gruppo Ceramiche Gresmalt, Via Mosca 4, 41049 Sassuolo, Italy

**Keywords:** Decision making, Economic hermeneutics, Flexibility, Interpretative analysis, Resilience, Risk management, Sustainable development, D81, L61, L21, L25, D24, Q01

## Abstract

Risk management plays a key role in uncertain times, preventing corporations from acting rashly and incorrectly, allowing them to become flexible and resilient. A global turbulence such as the COVID-19 pandemic has had a strong impact on individual companies and entire economic sectors, raising the question of whether a paradigm shift is necessary, in order to enable a new cycle of development that is much environmentally, socially and economically sustainable. This environmental and socio-economic context of profound uncertainty forces organizations to consider more carefully the risk factors affecting their business continuity, as well as how these factors relate to sustainability issues. However, there is a gap in knowledge about how risk management systems relate to sustainability management systems, and how both of them exert influence on business performance, especially from a theoretical point of view. The aim of this study is to address this gap, by developing a new interpretative framework for the analysis of risk management strategies in organizations. This approach has been identified in economic hermeneutics as an innovative methodological tool to improve the knowledge of risk and design the most appropriate management strategies. The paper provides two main results: the first one is the construction of a theoretical model that relates risk management to sustainability management; the second one is an operational framework of multidimensional risk assessment useful for analysis at different levels (business, competitive scenario and system). Finally, the model also makes it possible to carry out a sustainability assessment through risk evaluation in the perspective of the sustainable development goals.

## Introduction

Interest in risk identification has existed since ancient times, stemming from the need to prevent events unfavorable to the well-being of humanity (Crockford 1982). Since then, the development of risk management and related key functions has evolved over the years (Biolcheva 2020). Nowadays organizations carry out their activities within complex socio-economic scenarios in which it is essential to implement risk monitoring strategies. Business activity has always been characterized by an intrinsic link with risk (Osuszek and Ledzianowski 2020; Tiwari and Suresha 2021). However, despite the importance of this link, risk within the company has long been a marginal factor in the conduct of business. This has resulted in the mere operational implementation of measures, often separate from each other, aimed at mitigating the effects of an unexpected event, primarily on a financial level. With this type of approach, the risk assessment is partial because it does not fully consider the effects that critical events may have on the organization as a whole (De Smidt and Botzen 2018).

Social sciences, and especially anthropology, have provided the basis for overcoming this limited view of risk as they allowed to create the awareness that every stakeholder who is related to the organization has a different perception of risk (Boholm 2015). Consequently, it is necessary to adopt a holistic approach that includes each vision, also applying different assessment methods to achieve an integrated risk management strategy (Yoon et al. 2018). In social sciences, the concepts of risk and uncertainty refer to the possibility of unforeseeable events occurring, which can change the results of human activity in unexpected ways. According to the classic distinction between risk and uncertainty (Sakai 2019), a situation can be defined as risky when the probability of unexpected events occurring is known and objectively determinable. The risk is modified only because of a change in the objective parameters linked to the event or which depend on the environment or the economic system. The risk is contrasted with the uncertainty that arises when events cannot be classified with objective probabilities, but instead are linked to subjective probabilities, in the form of degrees of belief of economic agents. Uncertainty therefore expresses the perception that they have of the changing structures of the economy (Rogova and Ilin 2019). Thus, in social sciences, the distinction between a risky and an uncertain event is controversial as it is a consequence of the different way of observing phenomena and the possibility to objectively predict, or not, that they may occur (Zinn 2009; Shou and Olney 2020).

In addition to day-to-day risk management, emergency situations such as COVID-19 represent moments of disruption without precedent in economic, social, environmental, and geopolitical order at a global level (Fakhruddin et al. 2020; Ufua et al. 2021). The unforeseen crisis, which is prolonged over time, radically changes the operating scenario and the competitive environment of companies (Santibanez González et al. 2019). Individuals and organizations need security measures to contrast and mitigate the effects of the permanent risk situation. The pandemic state has also shown how ecosystems can regenerate themselves, even briefly, if companies, organizations, institutions, and individuals are able to rethink themselves in a resilient (D’Adamo et al. 2020a) and flexible way (Shukla et al. 2019; Sushil 2015). Several scholars have pointed out that the relationships between risk management strategies and sustainability management procedures, as well as how their interaction affects the performance of companies, have not yet been adequately investigated (Hallikas et al. 2020; Shad et al. 2019). This calls for the design of new models of economic, social, and environmental management that will better leverage the concept of sustainability and sustainable development, bringing people back to the center of all strategic thinking.

Therefore, this study seeks to fill the gaps highlighted in the literature, previewed above and discussed in the next section, by approaching the following research questions:

**RQ1:** How can an organization integrate the technical operational attribute of a risk with the strategic corporate attribute?

**RQ2:** Is it possible to design a sustainability-based risk management system and vice versa, a risk-based sustainability management system?

**RQ3:** How can an organization convert a risk management system into a competitive lever for sustainable growth?

This paper is structured as follows. After the introduction that introduces the synopsis of the study, the methodology section outlines how the research was carried out. The literature analysis then leads to the elaboration of a theoretical background, aimed at defining the concept of risk and establishing its different types. The construction of a conceptual model follows, laying the foundation for the development of a multidimensional risk assessment framework that includes both the pillars of sustainability and the technological dimension. Finally, the results are discussed, and limitations and future lines of research are addressed.

## Methodological Issue

### Research Aims

The aim of this research is to explore the relationships that exist between the sources of risks, and the potential impacts they may have on business operations. In an organizational context, risk management systems provide for the direct involvement of a technical specialist for each category of risk, who is tasked with identifying, measuring, and mitigating its negative effect. In this way, risk management is dealt with primarily on a technical level, without fully considering the implications that the occurrence of critical situations can have on organizations. The ineffectiveness of this management process has been demonstrated by both the past financial crisis of 2008 and the more recent pandemic crisis of 2020. In both cases, there was a lack of convergence between the risk management system and corporate strategy due to the lack of involvement of top management in decision-making processes relating to risks, having delegated all risk assessment and mitigation strategies to technical experts in each area. In addition, the aforementioned crises have stimulated the attention of both public authorities and companies from the sphere of economic–financial risk, towards trend-topics linked to the environment, in particular climate change, and the social dimension. However, this new sensitivity towards the so-called emerging risks has been limited to including the new risk categories in the conventional risk management framework, failing to grasp the opportunity to integrate risk management and sustainability management systems into a single strategic tool for the growth of companies towards sustainable development.

### Research Methodology

This study uses both analytical and empirical research methodologies in a hybrid approach (Ourston and Mooney 1994). The analytical research initially relied on an inductive content analysis of the literature to identify variables and theoretical constructs, necessary to build an explanatory conceptual model of the state of the art on the topic of risk management (Kaihlanen et al. 2018). Also, as part of the analytical research, the theorization of the concepts extracted from the literature allowed to design an explanatory and multidimensional matrix for sustainable risk management, employing abductive inference (Fei 2018). The explanatory matrix then provided the baseline for building a sustainability-based risk assessment framework following a hermeneutic interpretive approach (Miller et al. 2018). It proved to be particularly suitable to apply a holistic view to risk management and to allow to quickly change the perspective of analysis (from parts to whole, from micro to macro, from past to future) in an iterative mechanism (Settembre Blundo et al. 2019a). Finally, the risk assessment framework has been empirically validated applying it to the specific case study (Ridder 2017) of an Italian company producing ceramic tiles in the porcelain stoneware type (Biasini et al. 2002; Conte et al. 2020). This allowed carrying out both a retrospective and a prospective analysis drafting three scenarios: past, present and future.

## Theoretical Framework

We used a critical review method (Snyder 2019) to analyze and synthesize the literature, laying the foundation for the construction of a conceptual model adopting an interpretative approach (Hanafizadeh and Nik 2020).

### Risk Definition Background

The origins of the term risk seem to be lost in time, and the existence of studies that account for its use throughout history is unknown (Luhmann 1991). For Giddens (1994), the notion of risk appears in European thought around the nineteenth century with the English word that used to be spelled in its French version, “*risqué*”. For some time, French spelling continued to be used in conjunction with the new anglicized word “*risk*,” which began to be used in the field of insurance. Hansson (1989) argues that, in the scientific use of the term, risk is considered as a one-dimensional concept that refers to a numerical probability value, while in popular use it has many and more varied meanings. In the meantime, from an academic point of view, it has not been possible to establish a unitary definition of risk and even less to develop a coherent theory of risk (Crovini 2019). While the risk literature provides several classifications of the term itself, two general orientations can be identified from academic study. On the one hand, positivist approaches, in line with the natural sciences that work with quantifiable data and facts (O’Donnell et al. 2013). On the other hand, there are interpretative (Organ and Stapleton 2016) or hermeneutical (Kristensen et al. 2013) approaches that focus on the more qualitative aspects of the concept. Thus, we propose that:

**Proposition 1 (P1):**

The concept of risk does not have a unified and shared definition and, from the methodological point of view, both quantitative and qualitative approaches are used.

### Sociological Concept of Risk

In approaches relevant to sociology, the issue of risk is not raised as a purely technical issue that takes on the characteristics of social problems. The different sociological perspectives, in general, criticize the rationalist conception of risk and emphasize that there is a social construction of risk (Farrás et al. 2001). From a sociological perspective, different social groups develop different conceptions of the seriousness and acceptability of different risk situations, as well as of the preceding responses to each of these conditions. Such patterns are governed by the usual procedures of socialization and economic, political, and cultural factors rather than by the calculation of probabilities. Consequently, it is possible to state that there will be a differentiation between the perception of risk and the objective risk (Gordy 2016). Risk is intrinsically linked to a social or individual decision. The origin of the risk (and therefore of the subsequent changes triggered) no longer lies in an external or independent will; on the contrary, the responsibility for such a panorama is attributed to decisions taken and preferred choices. In line with some of the risk society approaches of the German sociologist Ulrick Beck (2012), the question is not so much whether contemporary hazards are more or less serious than those of the past, but that today hazards are usually attributed to human actions and decisions and are therefore given the form of risks.

Risks then emerge as a result of socio-cultural procedures that serve certain social and political functions, with the origin and its social consequences being studied, along with its symbolic use, the way in which faults and responsibilities are attributed, the role of experts and knowledge in their management, their unequal distribution, associated conflicts, or their relationship with the processes of modernization and globalization. From the sociological point of view, risk is defined subjectively by the affected subjects; therefore, there is an implicit element of cognition and individual perception. However, it is perfectly possible to obtain a certain consistency between the different definitions of individuals, to such a degree that the norms and lifestyles, and the effects of an event, are similar (Lucini 2014). On the other hand, risk is not only understood in terms of damage to property but also in terms of the discontinuity generated during daily life. In this sense, risk is viewed from the perspective of both individual well-being and the safety of family, friends and community partners (McIntosh et al. 2016; Zwetsloot et al. 2017). In order to understand risk, social scientists must therefore integrate the concepts of property damage and threats to living conditions. Thus, we propose that:

**Proposition 2 (P2):** The sociological approach to risk considers both the objective rational evaluation and the emotional and subjective perception, highlighting the positive or negative correlations between risks and benefits of an individual or collective activity.

### Business Concept of Risk

Business risk is based on the probabilistic nature of the firm's activities and the relative situational uncertainty in which they are conducted (Semenets 2019). These activities are constantly evolving and depend not only on circumstances that are often uncontrollable, including the environment, labor costs, raw material prices, technology, buyer behavior, competitors, regulatory and tax regime, but also on management choices (Hannabuss 2016). Therefore, business activity is necessarily accompanied by a level of uncertainty that defines the need to choose between different alternatives and to make decisions. It can be said that uncertainty dominates the whole life of the company in every phase and that risk can therefore be considered a component of the company itself, since it considers the possibility that the company does not have a stable ability over time to remunerate its productive factors (Elkhal 2019). This risk is therefore to be understood as the inability of the company to meet its industrial costs with income. The knowledge and management of business risk therefore becomes necessary for the functioning of the company in order to try and protect and safeguard the entrepreneur, shareholders and employees.

Nowadays companies are exposed to various risks that can affect their operations and can lead to a loss of customers and markets, reduced profits, machinery failures or a lack of liquidity. For this reason, it is necessary to put in place a risk management plan, which is a set of activities, methodologies and coordinated resources focused on identifying possible internal and external risks that may affect the company, in order to take the necessary measures and ensure its safety and integrity (Anderson 2013). This process of planning, organizing, managing and controlling a company's activities to minimize the effects of risk on capital and profits is often called Enterprise Risk Management (ERM), (Anton et al. 2020). Many scholars and practitioners have different views on risk classification systems and what types of risk to include or not in the ERM. These differences arise from the varied contribution that actors inside and outside the company make to the creation and evolution of the risk system over time. According to Dudin et al. (2016), two main types of risks (internal and external ones) can be distinguished, as well as some key types that must be controlled and managed by the company's management team in order to avoid threats of loss or impediments to the achievement of business goals. Thus, we propose that:

**Proposition 3 (P3):** Business risk is the set of possible negative effects, as well as potentially positive effects, that occur in a company due to an unexpected event of a technological, economic, financial, asset or reputation nature.

#### Internal Business Risks

Internal risks are those business risks that depend on the management of the company itself, both at a general level and in each of its functions and departments (Kiradoo 2019). Among the types of internal business risks, we can find the following:

- Operational risks. Operational risk is the possibility of losses to a company due to human error, process failures, inadequate technology and even the occurrence of unforeseen external events. This definition of risk includes legal risk, which is the risk arising from any defect in contracts entered by the institution, and penalties or compensation arising from damage to third parties (Naude and Chiweshe 2017). Systematic and reputation risks, as well as losses caused by changes in the political, economic, and social context, are excluded. Consequently, Operational Risk Management (ORM) is associated with operational processes, their elements, and results (Araz et al. 2020). In addition, operational risk directly affects the company's assets, depending on the severity of the event. The human resources factor is focused on financial losses associated with human errors of workers, partners, or managers such as: negligence, fraud, sabotage, theft, industrial espionage or money laundering, labor disputes. Losses resulting from a lack of clear specifications for the employment of personnel, or inadequate skills and/or training may also be included (Becker and Smidt 2016). The internal processes factor identifies losses related to the inadequate design of structures and procedures within the company that could cause inefficiency and deficient performance (insufficient evaluation of contracts and operations, failure to meet deadlines and budgets, errors in transactions or accounting information) (Blunden and Thirlwell 2012).
- Internal financial risks. This category includes all business risks with a strong component of financial sources that can be controlled directly by the company. Credit risk can cause a series of internal financial problems, one of which is mainly the lack of liquidity and the possibility that credits may not be recoverable. Liquidity risk prevents the company from satisfying its obligations to third parties. For example, companies may extend customer credit lines or accumulate debts with suppliers (Bhunia and Mukhuti 2012).
- Marketing risks. They include all those risks that occur in the procurement of inputs and the sale of the finished product. The high percentage of dependence on a few customers and/or suppliers represents a risk factor. The loss of one of them due to a lack of quality, rise in price, service failure or any other reason may result in a serious deterioration of profitability. Therefore, companies should aim at diversification of both suppliers and customers (Tkachenko et al. 2019).
- Occupational risks. They are defined as the hazards that exist in a specific occupation and professional activity, as well as in the environment or workplace, which can cause accidents or any type of accident that can lead to damage or health problems, both physical and psychological (Băbuţ and Moraru 2018). Risk factors are directly related to or dependent on the safety conditions that apply in the workplace, so occupational risks are often included among social risks, enterprise-wide (Schömann et al. 2006).
- Strategic risks. They refer to the possibility that a company is outperformed by the competition in its capacity for innovation once it has consolidated its position in its sector. The maintenance and growth of profitability should be supported by continuous innovation of processes, products, and the business model in order to attract new customers and maintain high retention rates. Otherwise, companies may lag behind their competitors who continue to improve (Ennouri 2013).
- Reputational risks. Businesses work hard to build a solid reputation that attracts customers and wins their trust. However, a dissatisfied customer, defective product or lawsuit can threaten a company's image. It is therefore possible to consider reputational risk as the possibility of spreading negative information and stereotypes (whether or not they are true) about a company's activities, which may compromise its trust, social credibility, competitiveness, and reliability. The reputational risk can be considered a second level risk since it is intricately linked to other risk factors. Businesses should use a reputation management strategy to constantly monitor and respond to stakeholders' opinions (Glickman 2014). Corporate Reputation, understood as the set of complex relationships between corporations and stakeholders, is manifested in Corporate Social Responsibility (CSR), this risk can be seen as an emerging reputational risk and not only internal to the company. The change in the stakeholders' scale of values, incorporating environmental concerns, conditions this hybrid character. In fact, if companies do not communicate their commitment to corporate social responsibility or do not do so in an adequate and carefully planned manner, they risk seeing their reputation seriously damaged, exposing themselves to criticism and public condemnation by stakeholders (Pérez-Cornejo et al. 2019).

Thus, we propose that:

**Proposition 4 (P4):** Internal business risks arise during the normal operations of the company, so they can be predicted with some reliability by management, mitigating their effects.

#### External Business Risks

External risks are those events that occur without the direct control of the company because they come from the external environment (Belinskaja and Velickiene 2015). However, like internal risks, they directly or indirectly influence or condition business operations and pose a threat to development. The firm cannot avoid these circumstances, but it can try to ensure that they affect it as little as possible when they occur.

- External financial risks. Market risk and trade credit risk are external financial risks. Market risk refers to changes in the value of an instrument or portfolio of financial instruments linked to unexpected changes in market conditions that affect the value of a company's assets and liabilities. It includes exchange rate risks arising from fluctuations in the prices of different currencies (due to supply and demand on the international market and government decisions), and interest rate risk which is a consequence of their own volatility. Trade credit risk, on the other hand, is due to a counterparty's failure to comply with payment obligations under the contract (Noor and Abdalla 2014).
- Economic risks. They refer to those changes within the economy that can have an impact on finances, the availability of capital and obstacles to access competition. The economy is constantly changing and moves through economic cycles, whether it is in recession or expansion. It is above all the economic cycles in recession that can pose a major threat to the company. Some changes, such as lowering wholesale prices, can lead to increased profits, while others can slow down the growth of companies, such as rising interest rates (Atanasov and Nitschka 2017). This type of risk affects the stability that is created between costs and revenues and therefore has significant impact on the effects of the income produced by the firm. Therefore, it is a factor that needs to be analyzed very scrupulously as it affects the skills and responsibilities of top management and therefore has a significant impact on the performance of the business (Cooper and Jarre 2017).
- Compliance risks. Regardless of industry, companies must follow laws and regulations that restrict their activities (Esayas and Mahler 2015). Therefore, compliance refers to the risk of incurring judicial or administrative sanctions, significant financial losses or damage to reputation as a result of violations of mandatory rules (law or regulations) or self-regulation (e.g., articles of association, codes of conduct, codes of self-discipline), (Nicolas and May 2017; Shivaani 2018). The management of compliance risks helps companies to promote their ethical values by improving relationships with customers, to protect administrators from possible personal liability and to align employees' behavior (Tams and Gentile 2020).
- Technological risks. These are risks associated with technological innovation processes that are potentially disruptive and forever change the way things are done (Schuh et al. 2020). The new technologies usually increase competitiveness by reducing costs, but a significant investment may not have the time to be amortized if new innovations follow one another quickly, making the technologies recently adopted obsolete (Birkel et al. 2019). In addition, the use of a cutting-edge technology can make the firm dependent on one or a few suppliers (Pellicelli et al. 2019). Finally, not keeping up with technology leads to a loss of competitiveness. Competitors who are more innovation-oriented will offer better products at a better price by taking advantage of the newer, more efficient and cheaper technologies (Li et al. 2016).
- Geopolitical risks. These are risks that arise from the political conditions in the country in which the company operates (Leitner 2016) and can be of two types: governmental and legal. The former includes all risks that are the result of actions implemented by local institutions, for example a change in government or a change in trade policies (John and Lawton 2018). The latter includes acts outside the law, such as insurrection, civil war or acts of terrorism or sabotage (Webb 2012). These risks produce legal or regulatory obstacles that may hinder a company's activities in a particular region.

Thus, we propose that:

**Proposition 5 (P5):** External business risks arise as a consequence of the general and competitive context in which the company operates and the ways in which it is forced to adapt to these conditions. Therefore, management cannot easily control them.

### Emerging Risks

In addition to those described above, a new category of risks is taking hold, characterized by continuous evolution and a greater degree of uncertainty due to the lack of historical data that can describe them (Cantonnet et al. 2019). These emerging risks derive from the most recent scientific–technological, sociopolitical, or regulatory changes that can create discontinuity in the life of companies. They can be considered new because they are risks that did not previously exist and are caused by new processes, technologies, or social and organizational changes, or by changes in social and public perception (Henne and Wenzel 2020). They can also arise from a known problem if new scientific knowledge identifies the problem as a new risk.

Thus, we propose that:

**Proposition 6 (P6):** Emerging risks occur under new or unknown conditions that have not been sufficiently investigated and quantified yet have a high potential for impact.

#### Environmental Risks

The term environmental risk refers to the negative consequences for the integrity of the ecosystem that can derive from business activities: these dangers can arise either from sudden and/or accidental events (accidental pollution), or from the progressive accumulation of toxic residues and/or polluting actions that can manifest themselves in a slow and progressive manner (gradual pollution) (Krzemień et al 2016). Environmental risks now affect the competitiveness and profitability of companies, given the increasingly stringent regulations on the use and protection of natural resources. For some time now, economic science has been examining and studying them within the category of business risks. If optimized, they can become one of the main sources of return on capital and, therefore, a strategic area for the management of the company, for which tools and methodologies have already been developed according to a risk management approach (Huang and Li 2018).

The environmental risk for a company refers to the danger associated with the cost due to the careless management of the environmental aspects of the productive activities carried out. They can be caused either by the direct responsibility of a company (or factory), of its manager or by the actual owner, in relation to incorrect or inefficient procedures or procedures in violation of an environmental standard or related to an omission or inertia of these subjects (Dragomir. 2019). Including environmental risk within the company's strategy represents a cost for the company (cost of investments, cost of technologies, cost of the change in the objective of the production process, cost of modernization, etc.) (Stončiuvienė et al. 2019). However, the environment can generate a competitive advantage in terms of business processes because, on the one hand, it eliminates waste and inefficiencies, reducing emissions or the cost of waste disposal (D’Adamo et al. 2019; Wang 2019) and on the other, environmental management within business strategies stimulates the innovative potential of managing directors, pushing them to make a series of investments that, without the pressure of the environmental variable, they would not have decided to make (Ervin et al. 2013). Environmental risk management therefore refers to a complex social process that aims to reduce the uncertainty factors relating to the negative impacts that human activity can have on the territory and on society. Consequently, the management of this risk cannot be reduced to the idea of a single action but must refer to a process through which an organization becomes aware of the risk it faces, to know it and analyze it (Kas’yanov et al. 2018). On this basis, with a strategic approach, the options, and priorities in terms of reducing environmental criticalities will have to be considered, the resources available to address them will have to be assessed, the implementing procedures will have to be defined and a decision will have to be taken to apply them. Therefore, corporate governance cannot ignore the management of the environmental risk associated with products, services and processes, in a social scenario in which the orientation towards sustainable development and pollution prevention must become an opportunity constraint and also a distinctive competence of the company in communicating with all stakeholders (customers, shareholders, institutions, the community, the financial world) (Helfaya and Moussa 2017).

Thus, we propose that:

**Proposition 7 (P7):** Environmental risk concerns the implications for a company's competitiveness of environmental damage caused by its activities. These effects can be both negative (increased costs) and positive (increased efficiency).

#### Supply Chain Risks

In general terms, a supply chain is the network that includes all the operators, organizations, resources, activities and technologies involved in the creation and sale of a product: from the supplier to the delivery of the finished product to the end user (Tripathi and Gupta 2019). The strength of a supply chain corresponds to the strength of its weakest link, as variability increases with the considerable number of processes that go to make it up. It is therefore necessary to identify potential breakpoints with action and prevention plans (Bevilacqua et al. 2018).

There are at least two risk factors: the bullwhip effect and uncertainty (Mangla et al. 2014). The bullwhip effect, also known as the Forrester effect (Naim et al. 2017), is the main cause of inefficiency within supply chains. It consists of an unpredictable and unjustified increase in demand found upstream in the supply chain. So, while consumer demand for a product tends to be stable and predictable, moving up the value chain the same demand is increasingly amplified and unstable. This risk is produced because the various actors of the supply chain use the data provided by the suppliers as an indicator of the level of demand, instead of relying on the final customer (Ma et al. 2019). The bullwhip effect is generated not only by the distortions of the demand, but also by the tendency of the single decision maker to optimize locally their own link, missing the opportunity to do likewise along the entire supply chain. Uncertainty, on the other hand, is the result of the natural time gap between supply and demand, accentuated by the lengthening and globalization of supply chains as well as the shortening of the product life cycle. This inability to match supply and demand generates, on the one hand, a loss of sales if supply in the supply chain is lower than demand and, on the other, obsolete or unsold products if it is not (Haines et al. 2017).

Both factors can generate known and unknown risks (Farahbod and Varzandeh 2018). Known risks arise from interactions between the actors in the chain and are also estimable and potentially predictable based on historical information. Risks that are unknown or lacking in historical information are due to unforeseen variations along the chain, erratic supply and demand behaviors, natural phenomena, or other types such as changes in geopolitical conditions. It is therefore clear that the complexity of the supply chains, in which the interconnections between the actors multiply, does not allow to evaluate the risks individually because a variation in one part of the system inevitably rebounds on the other parts, also causing business disruptions. To optimize the performance of a supply chain, it is consequently necessary to adopt an integrated and systemic approach with the aim of controlling risk exposure and reducing its negative impact on performance (Heckmann et al. 2015).

Thus, we propose that:

**Proposition 8 (P8):** Supply chain risk is an important part of running a business because it encompasses the procurement and distribution processes that together affect a company's operations. Reducing the risk of supply chain disruption requires a collaborative and proactive approach with all stakeholders.

#### Cyber Risks

Today's societies live on a constant path toward the digitization of information (Royakkers et al. 2018). Companies have had to reorganize their processes to adapt to the spread of electronic devices for individual productivity, constantly connected to networks, as well as to the success of the Internet of Things and Industry 4.0 technologies that have transformed production processes in the manufacturing sector (Kliestik et al. 2020). All the advantages that this digital transformation brings, however, are associated with a series of threats that jeopardize the security of systems and compromise the privacy of information (Alani and Alloghani 2019). In this digital environment, companies must analyze cyber risks and take measures to prevent them or mitigate their negative effects (Lezzi et al. 2018).

Cyber risk management seeks to ensure the integrity, confidentiality and accessibility of the entirety of an organization's information technology data, a task that is particularly important to businesses, so much so that the most modern management approaches consider cybersecurity to be a necessary requirement to ensure the proper operation of all business processes (Eling 2020). For this reason, cybersecurity has become a cross-cutting issue that encompasses all the distinct functions of organizations (Bharathi 2017). These threats to enterprise IT assets are primarily related to human resources, natural events or technical failures. A proper cyber risk identification process requires identifying all those informative assets that have some value to the organization such as associating the relevant threats with the identified assets, determining the vulnerabilities that can be exploited by these threats or identifying the impact that a loss of confidentiality, integrity and availability could have on each asset (Khodabakhsh et al. 2020).

Assessing cyber risk is an extremely complex activity because the probability of occurrence and the possible negative consequences that such an event could entail are difficult to determine. Another critical factor for companies is the correct assessment of the damage resulting from an incident or an IT attack; there are few cases in which this assessment is feasible and objective (Amin 2019). Among these kinds of attacks, economic damage is more easily quantifiable, especially if the cyber attacks are frauds and extortions aimed at economic advantage, such as intrusions for espionage or interruption of services. In the age of social media, in addition to direct economic damage, image and reputational damage is also of great concern. A cyber attack or a loss of data constitutes damage that risks compromising the trust of consumers and creating harm beyond expectations (Dreyer et al. 2018). Finally, there is a third type of cyber risk, that of sanctions, linked to the regulations on data processing which can impose extremely high monetary fines for administrative violations resulting from the violation of legal obligations (Zerlang 2017). Vulnerability to cyber risk is influenced by the level of digital culture of companies (Kelchevskaya et al. 2019). The risks are more widespread among companies with high technological content, but which do not operate in the IT sector. These companies in fact, unlike low-tech companies, suffer, attacks but, unlike IT companies, they have not yet developed an adequate defense capability. For this type of company, the probability that an attack will lead to business interruption is higher.

Thus, we propose that:

**Proposition 9 (P9):** Cyber risk is defined as any risk of financial loss or reputational damage to an organization resulting from some type of failure of its information systems. Therefore, it is not just a technology risk, but it is really a risk to the business.

#### Global Health Emergency Risks or Pandemic Risks

Global epidemics and pandemics negatively impact the entire social, economic and financial landscape (Qiu et al. 2017). The two terms refer to the way in which an infectious disease spreads among the population. The difference therefore does not concern the severity of the disease, but its geographical spread. An epidemic is defined as a disease with a spread delimited in space and time, affecting several individuals far greater than would have been expected in that period and in that area (Green et al. 2002). When an epidemic is very widespread and spreads simultaneously in different countries and continents, it is known as a pandemic (Morens et al. 2009), as in the case of COVID-19. Pandemic risk was already considered one of the main threats to businesses before COVID-19. In fact, in 2007, the World Economic Forum (WEF) began to consider pandemic risk and the massive spread of infectious diseases as emerging risks in terms of impact. In the annual publications of the Global Risk Report, from 2007 to 2020, the WEF has always placed this type of risk among the top ten in terms of impact (WEF 2020). Global health risks have the potential to influence the activities of individuals, businesses, nations and societies through their negative effects, highlighting the vulnerabilities of globalized economies and health systems, affecting business operations, supply chain continuity and consumer behavior (Viscusi 2020).

The spread of the COVID-19 pandemic during the year 2020 demonstrated the inadequacy of traditional risk models that had been refined since the global financial crisis of 2008–2012. Predictive models based on the analysis of historical data were insufficient to handle the unprecedented effects of the pandemic with the sudden disruption of global supply chains and the sudden closure of cities and businesses (Obrenovic et al. 2020). In view of this, in terms of preventing and protecting the health of individuals and the economy, the greatest safeguards and solutions to implement them must be provided by governments and regulatory authorities (Ansell et al. 2020). With this in mind, preparing as best as possible for pandemic risk means investing resources and time in researching institutional framework, logistics and healthcare solutions (Pons et al. 2020). These investments are borne by present generations, but their fruits are likely to be reaped by future generations.

Pandemic risk can have far-reaching implications on companies, from employee health to business interruption, multiplying the likelihood of other economic/financial, social, environmental and health risks occurring simultaneously. For that reason, companies need to understand how events can impact their processes and how it is imperative to respond quickly, improving their resilience (Fadel et al. 2020). To do this, it is necessary for companies to analyze their strategic and operational risks from a new perspective, including those categories of social, health and environmental risks, which traditional risk management approaches classify as medium-level risks, with a low probability of occurrence but remarkably high impact. Health risk requires a different methodological approach to traditional models (Chondol et al. 2020). In fact, if we perform a risk analysis evaluating people as we traditionally do in the business world, we will consider the set of individuals as a single asset, to which we would associate average indicators to quantify threats and vulnerability: age, virus exposure, previous pathologies, habits, etc. If we consider the population as an individual element, we associate all threats and vulnerabilities to society, as well as impacts and probabilities (Asante-Duah 2017). Therefore, with this approach we make a general estimate that only allows us to verify whether a risk exists but without correctly identifying the groups most at risk and how best to use mitigation measures to help them. Not all people are equally vulnerable and not all social situations and operating environments are equally likely to spread infection, but distinct factors are combined (Amin et al. 2019). For this reason, even in businesses, health risk management and protection measures cannot be the same for different cases. The risk must be assessed in detail based on available information in order to ascertain where and how to implement measures.

Finally, actions aimed at preventing the risk of new pandemics are also part of actions to combat climate change. Several studies affirm that there is a connection between climate change and pandemics (Donati 2020; Mishra et al. 2020; Phillips et al. 2020; Piacentini et al. 2020; Taylor 2020), for example by increasing the probability of pathogen hopping between animal species, up to humans (Watts et al. 2020). Specularly, the impact of a global pandemic and the temporary reduction in pollution associated with the contraction of economic activities can overshadow the perception of environmental risk. In addition, nations engaged in repairing the economic and social damage of the pandemic may no longer have sufficient economic resources to address the substantial investments and costs necessary to meet the environmental challenge (Leal-Filho et al. 2020).

Thus, we propose that:

**Proposition 10 (P10):** The global health emergency risks represent extreme health events that trigger at great speed the occurrence of other operational, economic, environmental, and social risks. Such complexity requires a holistic view of the interconnectedness of natural and anthropogenic systems and the effects of their interaction.

### Building a Conceptual Model

At the conclusion of the literature review, in order to provide an integrated overview, the theoretical concepts that arose from the processing are schematized in Table 1, where the conceptual variables and the propositions that link them together are summarized.

**Table 1:** Overview of theoretical propositions and variables

No.	Propositions	Variable 1	Variable 2
P1	The concept of risk does not have a unified and shared definition and also from the methodological point of view, both quantitative and qualitative approaches are used	Risk	Paradigm:
*Positivist or interpretive*			
P2	The sociological approach to risk considers both the objective rational evaluation and the emotional and subjective perception, highlighting the positive or negative correlations between risks and benefits of an individual or collective activity	Society	Social risk
P3	Business risk is the set of possible negative effects, as well as potentially positive effects, that occur in a company due to an unexpected event of a technological, economic, financial, asset or reputation nature	Company	Business risk
P4	Internal business risks arise during the normal operations of the company, so they can be predicted with some reliability by management, mitigating their effects	Operations	Business risk
P5	External business risks arise as a consequence of the competitive context in which the company operates and the ways in which it is forced to adapt to these conditions. Therefore, management cannot easily control them	Competitive context	Business risk
P6	Emerging risks occur under new or unknown conditions that have not been sufficiently investigated and quantified, yet have a high potential for impact	Emerging risk	Society
P7	Environmental risk concerns the implications for a company's competitiveness of environmental damage caused by its activities. These effects can be both negative (increased costs) and positive (increased efficiency)	Environmental risk	Operations
P8	Supply chain risk is an important part of running a business because it encompasses the procurement and distribution processes that together affect a company's operations. Reducing the risk of supply chain disruption requires a collaborative and proactive approach with all stakeholders	Supply chain risk	Operations
P9	Cyber risk is defined as any risk of financial loss or reputational damage to an organization resulting from some type of failure of its information systems. Therefore, it is not just a technology risk, but it is really a risk to the business	Cyber risk	Operations
P10	The pandemic risks represent extreme health events that trigger at great speed the occurrence of other operational, economic, environmental, and social risks. Such complexity requires a holistic view of the interconnectedness of natural and anthropogenic systems and the effects of their interaction	Pandemic risks	Society

The P1 proposition underlines that there is not yet a clear and agreed definition of the concept of risk, just as there is not an unequivocal consensus on the methodological paradigm to be adopted in the field of risk management, as it can be both quantitative (positivist) and qualitative (interpretive) in nature. The social dimension of risk is described in proposition P2 by emphasizing the coexistence of both rational and objective perceptions and emotional and subjective perceptions, as well as the coexistence of the individual and collective dimensions of risk. This dual attribute of risk can be seen as the explanation for the adoption of the two different epistemologies: positivist and interpretive. The P3 proposition defines business risks as those unforeseen, positive, and negative events that impact on business operations. They can be due to factors internal to the company and controllable by management (proposition P4), or to factors arising from the competitive environment and therefore not directly controllable by the company (proposition P5).

“Emerging risks refer to threats that are perceived to be potentially significant, but which may not be fully understood or assessed, thus not allowing risk management options to be developed with confidence” Renn (2014: 114). Emerging risks are becoming progressively more challenging issues. Currently, new types of risks are emerging, in addition to those described that, as proposition P6 states, are still partly unknown despite having potentially dangerous impacts. Among them, environmental risk is one of the most important and, as specified by proposition P7, it is attributable to the damage (or benefits) to the environment and the local area caused by the activities of the business. Similarly, unforeseen events that may occur in sourcing and distribution processes can cause disruption to the supply chain with serious repercussions for business operations (proposition P8). The proposition P9 emphasizes that cyber risk, although seen as an emerging risk, can no longer be considered exclusively a technical risk, but rather a managerial risk, as it has a serious impact on business activities. Finally, health and pandemic risks represent an enabling and facilitating factor for the simultaneous occurrence of all other risks, demonstrating that the traditional approach to risk management is insufficient in these cases (proposition P8).

**Fig. 1:**
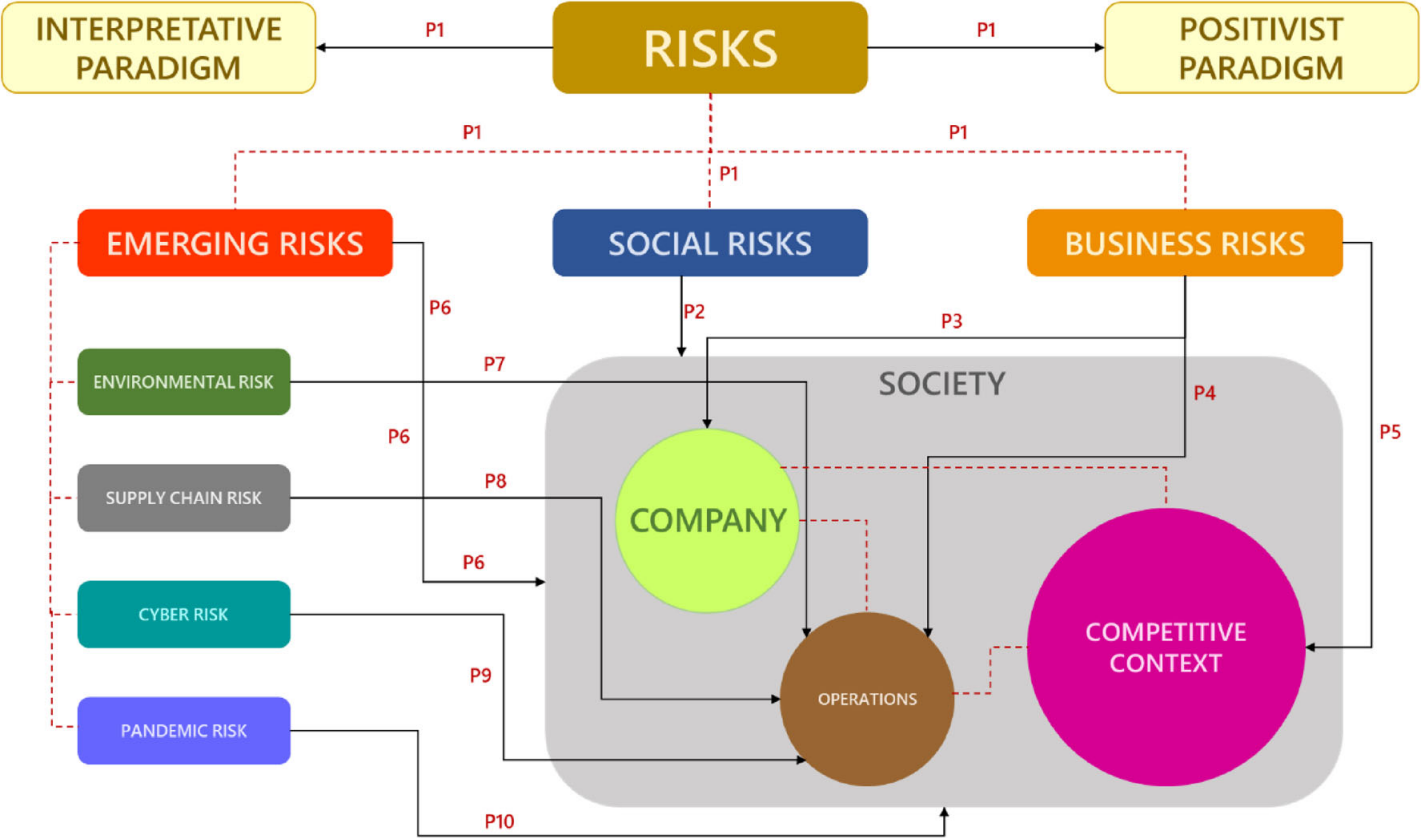
Conceptual model providing a descriptive overview of the concept of risk in management

A conceptual model (Fig. 1) is presented below which aims to constitute the argumentative framework of the theoretical framework discussed in paragraph 2. It represents an intuitive simplification of the reality related to risk management obtained through a process of abstraction of literature claims. The model relates the conceptual variables to each other through the statements of the propositions that are represented by the arrows and their respective numbers. The theorization highlights that the concept of risk can be explored with two different methodological approaches: quantitative positivist and qualitative interpretative (P1). From a managerial perspective, the different methodological approaches underlie the two main categories of risk for organizations: social risk and business risk (P1). Social risk is related to the society domain (P2), while business risk is related to the company (P3), its activities and operations (P4), and the competitive environment in which it operates (P5). The company domain, of course, also includes the enterprise and its operational environment. In addition to these two main risk categories, there are also so-called emerging risks (P1), which are related to issues that are new or not yet known to companies in the same depth as other risks. Emerging risks have an impact on society as a whole (P6) and therefore also on the life of companies. Environmental risks are among those most perceived at the corporate level (P7) because the issue of environmental protection is increasingly present on political and media agendas and is changing consumer purchasing criteria.

Concern about supply chain risk is growing significantly because the efficiency of the process of transferring a product from producer to customer determines the degree of competitive advantage of the entire supply chain and of individual companies (P8). In fact, the presence of relationships and interdependencies between different economic agents means that the performance of chain members, upstream and downstream of each firm, can significantly affect the overall efficiency and effectiveness of all the firms in the chain. Another new risk factor for companies is cybernetic (P9) which originates from the global space resulting from the interconnection of all the heterogeneous and interdependent networks made up of information processing systems and communication infrastructures. This cyberspace on which organizations depend is characterized by built-in technical and structural weaknesses that make it highly vulnerable to actions deliberately aimed at altering its functioning for fraudulent purposes. Finally, the health risk in general and specifically the pandemic risk are emerging risks which have long been underestimated, probably because in the traditional classification they have always been considered as second-degree risks due to the importance of other risks or disasters. Therefore, although it has a great impact on companies, they find themselves unprepared to mitigate its effects (P10).

The existing body of literature therefore highlights the inadequacy of the traditional approach to risk, as each category of risk is viewed and managed with specific tools, actions, and skills without considering the interdependencies between their various sources. Even the category of emerging risks appears to be completely outdated, given that the critical environmental, supply chain, cyber and health issues that have developed globally have had and continue to have a great impact on the lives of companies and their performance. It follows that even from the methodological point of view, the dualism between quantitative and qualitative methodologies is inappropriate to respond effectively to the challenge of increasing complexity. Based on these findings, in the following paragraph, an alternative approach to risk management in businesses is provided.

### Designing a Sustainable Risk Framework for Businesses

In order to offer an alternative to the current categorization of risks, a new framework has been developed (Table 2) which, based on the criterion of the level of analysis (micro, meso and macro), also offers a reading of risks in terms of sustainability, thanks to their connection with the sustainable development goals (SDGs). The matrix shown in Table 2 proposes the types of risk, identified in the analysis of the literature, aggregated through their level of occurrence, therefore on a micro-business scale, meso-competitive context and macro-global systemic scale (Serpa and Ferreira 2019).

**Table 2:**
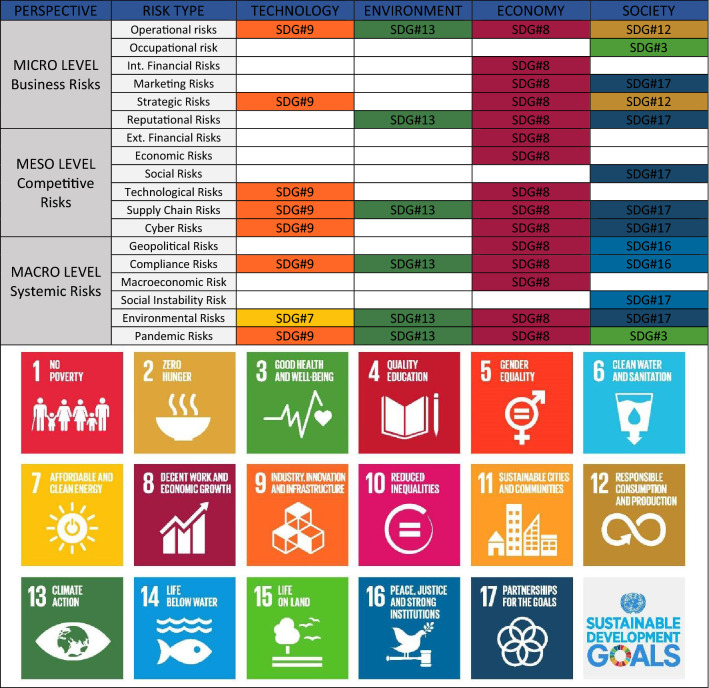
Sustainable multi-dimensional risks matrix with potential contribution to SDGs

**Table 3:** Framework for carrying out the interviews, adapted from García Muiña et al. (2019)

Business function	Job position
Board of directors	Chief Executive officer
Top management (C-level)	Chief financial officer
	B2B sales director
	B2C sales director
	Technical director
Management (B-level)	Procurement manager
	Sourcing manager
	Innovation manager
	Marketing manager
	Administrative manager
	Controller manager
	HR manager
	IT manager
	Credit manager
	Logistic manager
	Security manager
	Quality manager
	R&D manager
	Plant manager 1
	Plant manager 2
	Plant manager 3

This categorization, compared with that currently in use, makes it possible to better identify the sources of risk and to prepare contrast and mitigation actions that are more relevant to the domain to which they belong. In fact, a risk that may manifest itself by impacting on the company must be prevented and countered with direct internal actions taken by management using the organization's own resources. On the contrary, a systemic risk of a global nature sees the company committed to implementing only mitigation strategies, due to the fact that the origins of the risk can be identified outside its own operational perimeter. The intermediate meso-domain, which can be traced back to the competitive environment, requires a combined strategy between the firm and its supply chain partners, also considering the positions taken by political and economic–financial institutions. By adopting this type of segmentation, it is also intended to overcome the category of emerging risks, which can no longer be considered "new" by companies but, on the contrary, require in-depth knowledge of them to prepare and implement the most appropriate prevention, contrast and mitigation strategies. In addition, the so-called emerging risks can occur at company level, in a competitive and global context. Therefore, the response strategies of companies must be consistent with their originating source and with the specific situation in which they produce their impact.

The matrix in Table 2 also associates the different types of risk with sustainability and sustainable development, which are nowadays issues of central importance in business strategy (Galpin et al. 2015). The concept of sustainable development, initially associated with the environmental and social spheres, was then extended to the economic sphere, becoming the third founding element of the pillars of sustainability at the basis of Corporate Social Responsibility (Ye et al. 2020). Consequently, the efforts of control, evaluation and risk analysis of companies have also been mainly concentrated on the economic and financial sustainability of the business, leaving aside nonfinancial concerns. In addition, the emergence of Environmental Social and Governance (ESG) as criteria used for the evaluation of investments and activities of companies (Ortas et al. 2015) has enabled the assessment of corporate sustainability in a single integrated vision of economic, environmental and social aspects, including through the evolution of the concept of Enterprise Risk Management (ERM) into Sustainable Enterprise Risk Management (SERM) (Oduoza 2020). This approach aims to include in business reporting documents, in addition to financial risks, also environmental and social risks, expanding the types of risks to be included in the analysis. On the other hand, the opportunity offered by the Sustainable Enterprise Risk Management tool to carry out a comprehensive assessment of corporate sustainability by integrating environmental, social and economic assessment with risk assessment has not been fully exploited. Precisely to explore this possibility, the three pillars of sustainability (environment, economy and society) have been introduced into the matrix in Table 2, attaching them to a fourth pillar: technology. Technology is nowadays an area that surrounds and influences all business activities, including risk management, so it is impossible to address sustainable corporate management without also considering technological sustainability (D’Adamo et al. 2020b). It can be considered as the state of performance conformity of a process, product, or service that, in equilibrium with environmental and socioeconomic performance, establishes its suitability for use.

To strengthen the relationship between risk management and corporate sustainability assessment, each type of risk has been correlated with the sustainable development goals for each pillar of sustainability, in accordance with the 2030 Agenda (Tsalis et al. 2020). Therefore, the matrix in Table 2 offers a logical and conceptual framework to carry out risk assessment in terms of sustainable development, which, integrated with the classic tools of environmental (Ferrari et al. 2020), economic (Neugebauer et al. 2016) and social assessment, can complete the sustainability strategy both in a corporate (García Muiña et al. 2019) and competitive context (Settembre Blundo et al. 2019b), taking into account the global and systemic implications (Hosseini and Kaneko 2011) on corporate performance. With such integration, the long-term growth of the enterprise is consolidated as, from a sustainable development perspective, the monitoring of business operations is more effective and efficient due in part to how risk is managed.

### Elaborating an Interpretive Hermeneutics-based Risk Assessment Framework

In recent years, an attempt has been made to quantify risk, especially for management purposes, as the possible operational, economic, social and environmental consequences that may occur at a given place and time. However, risk has not been analyzed integrally, but rather piecemeal, according to the methodological perspective of each field of knowledge involved in its assessment (Soomro and Lai 2017). The lack of a holistic view of risk, i.e., a comprehensive and multidisciplinary assessment of it that would allow it to be broken down into its different components (Lacković et al. 2018), has manifested itself in conjunction with the occurrence of major global risks such as the COVID-19 pandemic emergency in the year 2020. Precisely in response to the new criticalities, a holistic view that addresses all dimensions of risk factors while also considering in a more systematic way the nonlinear relationships of environment parameters and complexity of social systems, could facilitate and orient the decision-making processes of organizations. The literature review has highlighted that the concept of risk is not always one-dimensional and objective since the same risk can mean different things to different people or in different contexts. For example, a risk that scientific experts objectively view as a product of nature, other individuals may view in relation to human decisions and vice versa. In this sense, risk assessments do not rely solely on empirical judgments, but also on constructed sociocultural notions that emphasize some aspects of the hazard and ignore others. For this reason, any epistemological approach to risk cannot be based exclusively on individual subjective assessment, but it is also necessary to consider the mechanisms of perception present in the social interaction of the individuals themselves. And it is precisely for this reason that the criteria of acceptability of risk are socially established, as are the principles of codification with which dangers are recognized and responsibilities assigned.

**Fig. 2:**
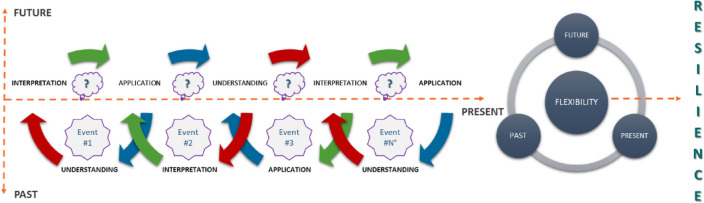
Hermeneutical approach to risk assessment, adapted from Hurmerinta et al. (2016)

Risk management is consequently not limited to a single event or circumstance but is instead a dynamic process that develops over time and permeates every aspect of the organization's resources and operations. It involves people at all levels and requires looking at the entire organization as a portfolio of risks. It occupies a place and takes on a strong importance within the broader definition of business management, as its function is to minimize the negative impact of losses on the organization. Therefore, interpretive hermeneutics (Farooq 2018) was adopted in this research to design a sustainable risk assessment framework, in order to obtain a risk assessment capable of integrating subjective and objective assessment, individual and collective dimensions, quantitative and qualitative data on the three levels of analysis (micro, meso and macro). This methodology is based on a holistic, inductive and idiographic approach, that is, it studies phenomena in their entirety and interpretations are developed from data and not from previous theories, focusing on the peculiarities of the specific case rather than the implementation of general rules and principles. This methodological approach, unlike others, is not linear because it is subject internally to changes that may occur during the analysis, becoming interactive with the research itself and the subjects that participate in it. The iterative process is identified as a hermeneutic circle (Rodighiero and Romele 2020) to indicate the dynamic relationship between the part and the whole: to understand each part, one observes the whole and to understand the whole, one observes its parts. In this iteration, the analysis of an event is conditioned by a pre-understanding based on a set of prior knowledge that determines the understanding of the present state. The understanding of an event is thus a historical fact determined by this incessant circular stratification of past experience and knowledge. In accordance, therefore, with Gadamer (1998), the hermeneutic circle has three main stages: understanding, interpretation, and application. The development of the dimension of time in the hermeneutic circle has been discussed by Hurmerinta et al. (2016), and following their approach, we can see that the understanding phase sinks into past experiences to interpret the present, projecting the analyst into a future perspective with the application phase. The schematization of the aforementioned iterative process based on the hermeneutic circle is illustrated in Fig. 2.

A risk analysis based on this model implies that the enterprise, for each category of risk related to an internal or external event, draws on its previous experience to read the present and design a counter and/or mitigation strategy for the future. In this way, the organization should be able to modify its behavior before, during and after a change that subjects it to stress. The reading of the past (understanding phase) corresponds to the capacity for anticipation: what are the foreseeable risks? How to prepare for them? Then the analysis of the present (interpretation phase) is achieved by monitoring current operations: how is the risk management system working? Finally, the projection toward future events (application phase) must help define the ability to react: will the organization be able to provide immediate responses to critical issues? The hermeneutic circle closes by returning to the past through learning: has what happened been incorporated into the company's know-how to increase its ability to respond? The iterative process, replicated for each category of damage across past, present, and future, crossing understanding, interpretation, and application, can improve the competitiveness of the firm by operating in two ways. On the one hand, by increasing organizational flexibility, i.e., the ability to respond effectively to both endogenous and exogenous critical situations, through adaptation to high-stress situations (Shukla et al. 2019). On the other, by stimulating organizational resilience, i.e., the agility to rapidly transform operations to deal with adverse situations, with a positive outlook (Miceli et al. 2021).

**Fig. 3:**
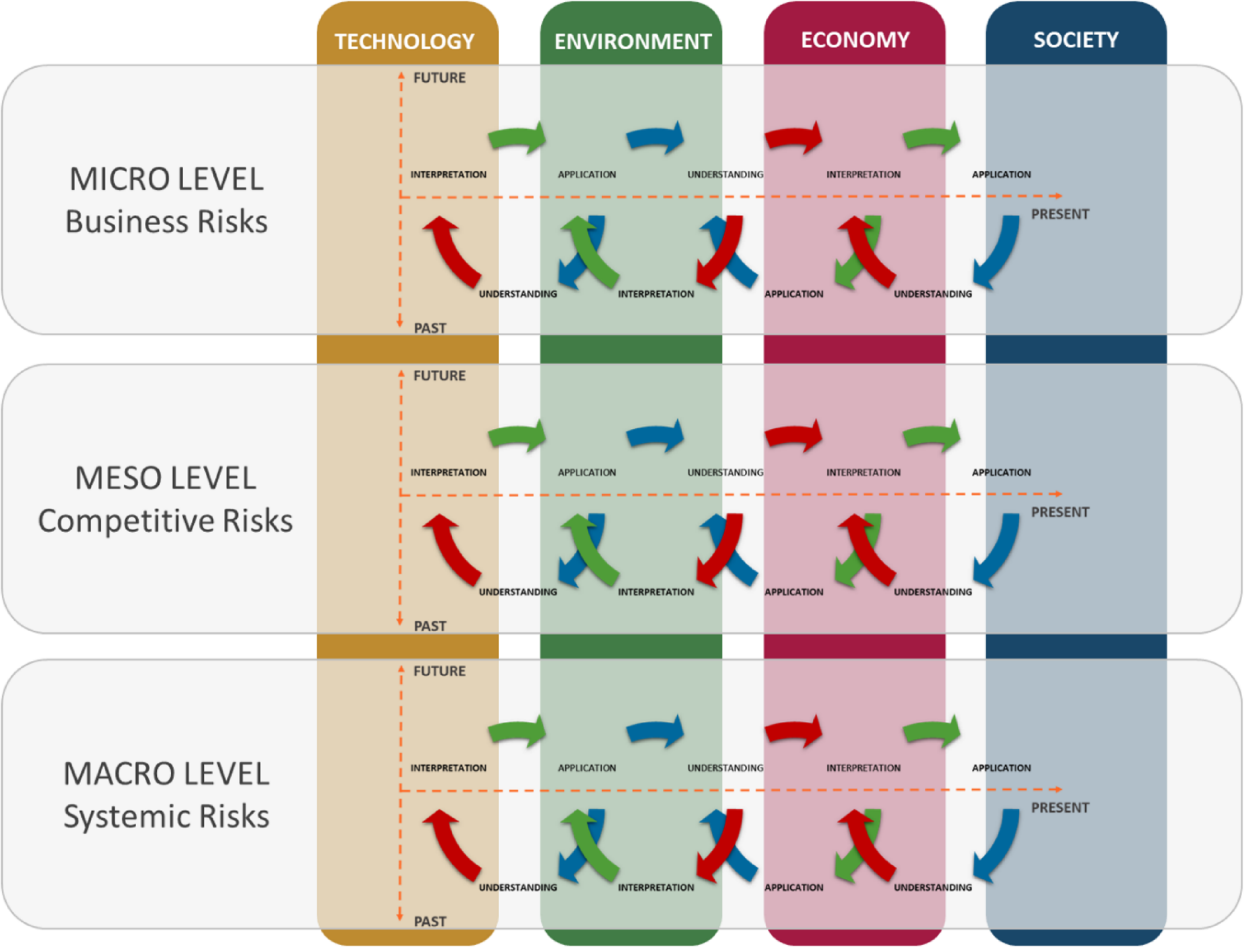
Flexible and resilience multidimensional approach to risk assessment

The hermeneutic approach to risk assessment, from a strategic flexibility and resilience perspective, was then integrated with the three pillars of sustainability (environment, economy, and society) associated with the technological dimension of sustainability itself, as shown in Fig. 3. Consistent with the matrix illustrated in Table 2, each type of risk categorized with the three levels of analysis (micro, meso and macro) is cross-referenced with the four dimensions of sustainability. This has the purpose of both deepening the degree of risk analysis and correlating the result of this assessment with the effects exerted by critical situations on each dimension of sustainability. In this way, the current paradigm is overturned, which also limits itself to classifying sustainability-related risks, while considering each risk as a key element in defining the sustainability of an organization from a sustainable development perspective. So, if we see sustainability as the pressure (impact) exerted by an organization on the context in which it operates, strategic flexibility and resilience will define the ability to respond adequately to the reactions of the context to these pressures. In addition, from the perspective of rational risk management, the company can use the proposed framework to decline resilience in three directions:

1. Analysis of the internal structure: *understanding past experience → risk prevention*.
2. Analysis of interdependencies with the context: *interpretation of the present reality → risk monitoring*.
3. Strategic design: *future application → organizational transformation and adaptation*.

## Results and Discussion

### Case Study

In order to empirically validate the sustainable risk assessment framework described in the previous paragraphs, the methodology of the explanatory single-case study was used (Nabhani et al. 2018). The company that is the subject of this case study is an Italian manufacturer of ceramic tiles that ranks one of the best performing (among the top 10) in the Italian ceramic sector. It represents a manufacturing excellence at European level that for years has focused its strategies not only on technological innovation but also on environmental sustainability (Ferrari et al. 2019). The sector is made up of 135 companies employing 19,318 people, which during 2019 produced 400.7 million square meters, such as to allow sales of 406.9 million square meters. Also, in 2019, the total turnover of Italian ceramic companies reached 5.34 billion euros, of which 4.5 billion (84%) came from exports (Confindustria Ceramica 2020).

The semistructured interview technique was applied to perform the sustainable enterprise risk assessment, replicating the procedure used by García Muiña et al., (2020) to map the key stakeholders of the same company that is the subject of this study. To this end, the authors selected twenty-one apex positions among the board of directors and top and middle management to conduct the interview, as shown in Table 3.

**Fig. 4:**
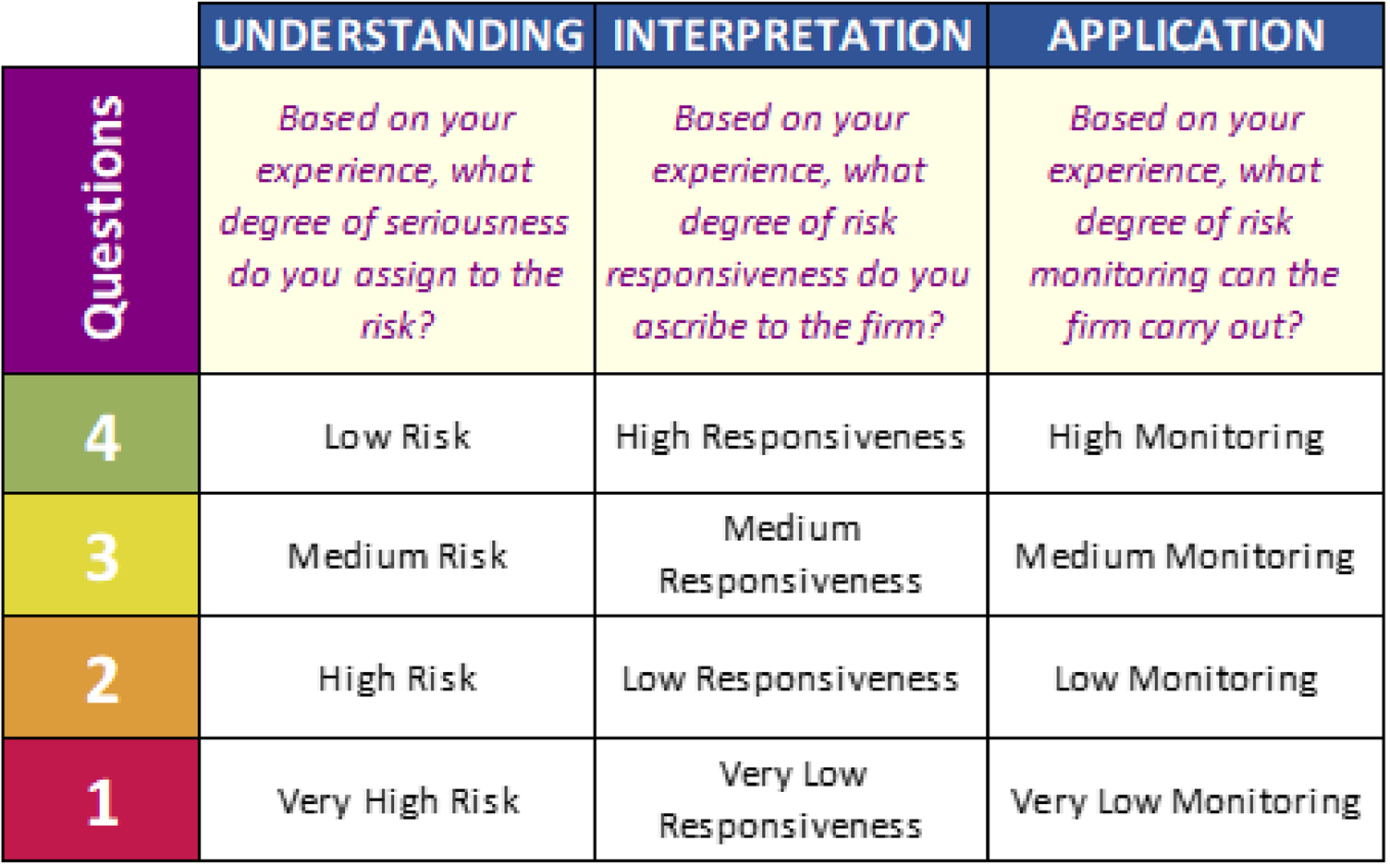
Likert-type rating scale adopted

Each of the selected managers was asked to assess the relevance of each type of risk already described in Table 2, employing the 4-point Likert rating scale (Croasmun and Ostrom 2011; Solke and Singh 2018), shown in Fig. 4.

The interviews were then checked and digitally transcribed for subsequent evaluation of the results (Medina-Salgado et al. 2021). Table 3 also shows the framework for conducting the interviews and the questions asked of the sampled company executives in order to capture the different awarenesses on the topic of risk in its multidimensionality (technology, environment, economy and society). For the comprehension phase of the hermeneutic circle, respondents were asked to express the degree of seriousness of the risk (from low to very high); for the interpretation phase, they were asked to express the degree of risk responsiveness of the company (from high to very low); finally, for the application phase, they were asked to indicate the degree of risk monitoring by the company (from high to very low). This procedure was replicated for each risk and for each dimension of sustainability, to carry out an in-depth analysis of the relationships between sources of risk and type of impact (technological, environmental, economic and social), both with respect to the company (micro-level) and to its competitive context (meso-level) and the global system (macro-level).

**Fig. 5:**
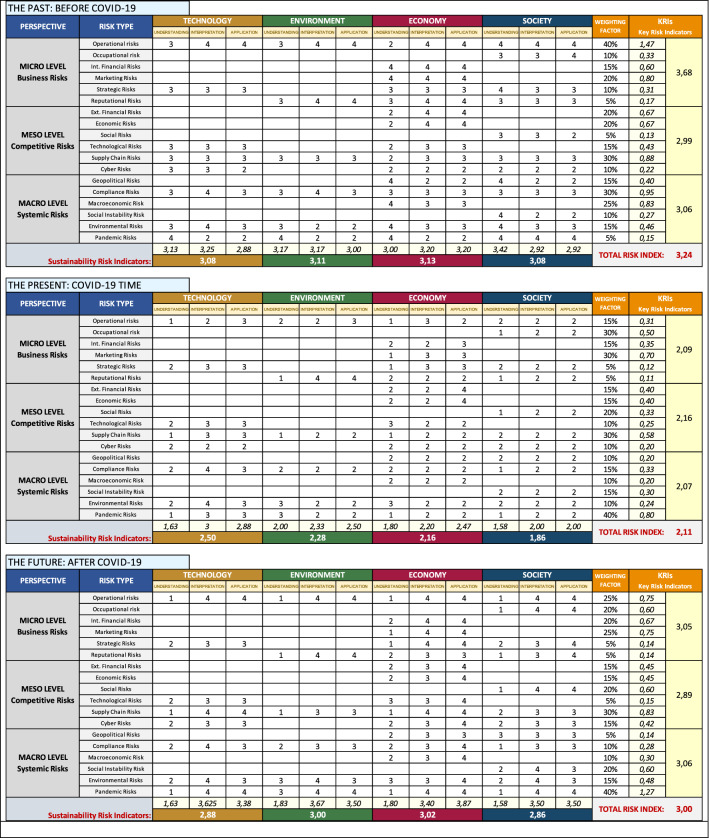
Framework for sustainability-based risk assessment

The analysis was conducted with three distinct time scenarios, taking the year 2020 as the time of disruptive change due to the COVID-19 pandemic (Biron et al. 2020; Mofijur et al. 2020). Respondents were then asked to perform the risk assessment described above in retrospective form by simulating a pre-pandemic situation (the past), then asked to repeat it by drawing the contemporary pandemic situation (the present) and a prospective post-pandemic situation (the future). The same panel of managers was then asked to assign a weighting factor to weight the relevance of each type of risk based on their own subjective perception. The results obtained for each scenario are shown in Fig. 5.

Regarding the Likert scale points collected from the interviews, the simple arithmetic mean was obtained for each type of risk and weighting was applied only to the result of this calculation (Naghshineh et al. 2020). The authors decided not to apply weighting factors for the four dimensions of sustainability (technology, environment, economy and society), or for the three steps of the hermeneutic circle (understanding, interpretation and application); similarly, neither were weighting criteria applied to the levels of analysis (micro, meso and macro). This is because the panel of experts consisted of a small number of people (21 middle and top managers), so weighting a few observations would have added further attributes of subjectivity to the results. The authors felt that the arithmetic mean across all observations could provide a more realistic picture of the actual risk culture present in the company. In this way, a set of key risk indicators (KRIs) was constructed, consisting of a weighted indicator for each type of risk, which when added together provided a level risk indicator (micro, meso and macro). Instead, the sum of all the KRIs provided a total risk index expressed by a point value consistent with the Likert scale employed. Finally, a sustainability risk indicator (SRI) for each pillar of sustainability was calculated through the arithmetic mean of the Likert points. In more complex case studies, where more individual observations were available, it would be possible to supplement the model proposed here with additional weighting criteria designed to focus attention on one more aspect of sustainability or a particular level of analysis.

The picture that emerges from the risk assessment clearly shows how, before the onset of the health criticality due to COVID-19, the company considered the pandemic risk low and its response capacity and monitoring criteria effective, at least in relation to the social pillar of sustainability. However, while the company viewed the pandemic risk to be always low, it ranked both its response capacity and monitoring to be low with respect to the impact of this risk on the technological, environmental and economic dimensions of sustainability. This result highlights a potential vulnerability of the organization, acceptable only in periods of stability such as the one considered. Precisely in that period, the company was mainly focused on paying attention to arising risks in the economic–financial dimension, leveraging its operational strength and financial solidity, characteristic of a manufacturing company (García Muiña et al. 2018). The risk assessment for this period therefore provides a Total KRI of 3.24 points, almost equally divided between the four pillars of sustainability (technology, environment, economy and society) equal to 3.08, 3.11, 3.13 and 3.08 points, respectively. The situation, therefore, which shows stable conditions, is of medium–low overall risk.

The occurrence of an event as serious and unexpected as the pandemic crisis of COVID-19 overturns the perception of risk by the company. This confirms the hypothesis that the pandemic risk, being global, enables the simultaneous occurrence of almost all other types of risk, also impacting on the company's strengths, namely its operating capacity and financial solidity. The total KRI falls by over a point, from 3.24 to 2.11, and the risk relating to social sustainability is reduced from 3.08 to 1.86 points. This shows how a health crisis makes social capital in general and human capital extremely vulnerable. Under these critical conditions, overall risk conditions decrease toward the high- to very-high-risk threshold.

Based on past experience, which has now become historical information and therefore a source of organizational learning, top and middle managers envisage the future post-pandemic scenario for the company, changing perspective from operational to strategic and vision from short to medium-long term. Keeping the level of pandemic risk high (1 point on the Likert scale) and aware of the impact that this source of risk exerts on all business operations, such as the continuity of supply chains, the panel of experts believes it is necessary to strengthen the capacity to respond to the multiplicity of risks and the monitoring actions. In this way, the total KRI should be reported close to the safety threshold represented by the 3 points of the Likert scale. Compared to the pre-pandemic scenario in the past, characterized in both cases by a low average risk index value, the company has changed its methods of perceiving risk by striving to grasp in depth the interdependencies between the various risk factors and the impacts they exercise not only on operational activities. With this assessment, which has been extended from the past to a future perspective, it has also been possible to measure the company's level of sustainability by using risk management as an interpretative key, measuring the state of equilibrium between the environmental, economic, social and technological pillars of sustainability.

## Conclusion

In economic contexts characterized by increasing complexity, risk seems to be an increasingly central concept in managerial practice, becoming the pivot of corporate action and the very foundation of entrepreneurship. The importance of achieving correct risk management as a generator of value in organizations justifies the interest that this subject has received from scholars. Risk assessment and risk management provide a useful tool in decision making, but there is still a need to achieve a higher scientific output (Aven 2016). Above all, the dimension of risk also hides, alongside potential and inevitable dangers, a series of opportunities which, if well integrated into business processes, can enable the reduction of the negative effects of critical situations and illuminate the road to recovery.

Therefore, as demanded by the current research, it is necessary to explore tools that could offer a multidimensional risk perspective with a more strategic rather than merely technical approach (RQ1), which could reflect the role of risk in corporate sustainability and vice versa (RQ2). With the conceptual model proposed in this paper, based on a systematic review of the literature and interpretive hermeneutics, it is thereby possible to integrate both the strategic and the technical operational attributes of risk. It offers a broad insight, both quantitatively and qualitatively, into the influence of risk on the competitive, corporate and social dynamics of the company. Based on this conceptual instrument, a concrete risk management tool is developed that relates the different sources of risk to the different aspects of sustainability. It allows to assess risks in terms of their influence on sustainability (a sustainability-based risk management system), and the priority of sustainability objectives can also be evaluated in terms of the magnitude of the potential risks that the company may face (risk-based sustainability management system). Thus, the second research question is answered. With respect to the last research question (RQ3), this tool, as the case study has shown, facilitates the monitoring of risks in different scenarios, which provides a better decision basis for anticipatory responses. All this will help to build adaptive capabilities that can evolve into a valuable competitive advantage.

Rethinking the risk management system can lead an organization to be more flexible and therefore resilient and proactive. This paper contributes to establishing a conceptual map of risk from different areas of knowledge. To this end, the main risks have been analyzed from a dual perspective. Firstly, whether the risks are related to factors which are external or internal to the organization. Secondly, according to the degree of uncertainty associated with the study of this analysis, by incorporating the so-called emerging risks, associated with a greater degree of uncertainty given the lack of historical data.

A second contribution of this research is related to the implementation of risk management. The sources of risk have changed and become more interconnected, making business contexts more complex. In addition, the areas of influence of business activities have expanded, giving rise to the concept of sustainability, to which risk management cannot be indifferent. Aspects such as flexibility and resilience are necessary to face uncertainty and current threats and require new, more comprehensive, and integrated approaches to risk. Given the importance of the application of sustainability principles in current business activity, the sustainability dimensions have been incorporated into the proposal of a risk analysis model in three possible scenarios. The analysis was conducted with three distinct time scenarios, taking the COVID-19 pandemic as the disruptive element (Mofijur et al. 2020). In the pandemic scenario, the company has changed its risk perception methods by seeking to recognize in depth the interdependencies between the different risk factors and the impacts they exert on operational activities. This study offers a framework for the management of business, competitive and systemic risks, which integrate into three perspectives—micro, meso and macro—for each sustainability dimension. The results support the importance of including a sustainable vision of risk management and allowing to quickly change the perspective of analysis.

The overview that emerges from risk assessment clearly shows how the company's perception of it changes with respect to factors that represent a dramatic break with the previous situation, such as the emergence of a global pandemic. In other words, global risks affect all other forms of risk, operational capacity and decrease financial strength.

### Implications for Researchers

Current research on risk has developed predominantly from a practical perspective. Therefore, there is a need for further contribution to the development of a theoretical framework as a basis for the development of different models. This paper contributes to this construction of a multidimensional theoretical framework. These perspectives enrich the framework by considering external and internal variables on the one hand and quantitative and qualitative variables on the other.

Moreover, the incorporation of the new types of emerging risk makes an additional contribution by enriching the existing studies up to this point. “*Emerging risk related to an activity when the background knowledge is weak but contains indications/justified beliefs that a new type of event could occur in the future and potentially have severe consequences”* (Flage and Aven 2015). The integration of this type of risk alongside classical risks provides a complete and more up-to-date theoretical framework. In short, the study builds a theoretical proposal for understanding risk in all its magnitude and suggests a model as a first step to investigate the different relationships between risks to which companies are subjected in complex environments. For example, environmental risks may be influenced by the different ethical perception of the company and may in turn have an amplifying effect on reputational risks depending on the geographical, social or competitive contexts in which the company operates. Cyber risks, on the other hand, could increase the company's vulnerability to other threats, again having an amplifying effect on other risks.

### Implications for Practitioners

Global and persistent crises such as the current pandemic have exposed some of the shortcomings of the traditional risk management approach. The lack of convergence with strategic direction and the treatment of risks from a fundamentally technical and isolated point of view have revealed the inefficiency of current risk assessment systems. The proposed model goes beyond the mere monetary quantification of different technical risks, improving the strategic selection and implementation process and thus contributing to the success of the organization. The implementation of such a tool can help the company to have a deep understanding of the risks it faces, the risks it internalizes and how it controls them at different levels of action from a dynamic outlook, also to capture new business perspectives (Polas and Raju 2021).

This proposal makes it possible to establish relationships between the sources of risk and sustainability in all its aspects. These linkages contribute to creating a map of vulnerabilities, exposures and hazards of the organization itself and its context in the pursuit of the objectives of sustainability and maintenance of the business "on-going", prompting top management to develop a more comprehensive strategic thinking that will be useful in the pursuing of capabilities related to anticipating and adapting to change and overcoming extraordinary wide-ranging events.

With a holistic view of each type of risk, the practitioners can have the tools to help manage them by mitigating their impact in the organizations. It is worth highlighting the need for managers in the different sectors to obtain guidelines for action in the current pandemic situation, due to the global socioeconomic impact of COVID-19 (Mofiju et al. 2020). A complete risk management function can be a value-adding resource in the strategic planning process.

The implications of the results obtained, in terms of risk assessment, are related to the development of organizational resilience to the high and diverse number of risks that currently must be dealt with. Just as there are many sources of possible risks, resilience is an organizational property that depends on the interaction between different factors in the system. The difference between the two approaches lies in the fact that in a resilience analysis, management does not ask the question of the cause of the unexpected event, whereas in a risk analysis and management, it is essential to identify the sources of disruption to business operations. Therefore, in business practice we can consider the sustainable risk assessment model as an operational management tool to build a more resilient organization.

Finally, the multidimensional sustainable risk assessment framework can be a useful tool for measuring progress in achieving the sustainable development goals. In fact, the key risk indicators can be weighted not only according to the sustainability pillars, but also to the SDGs associated with each type of risk. With this approach, it is possible to make the so-called SDGs-Washing risk (Munro 2020) visible. This is a reputational risk that arises when companies use the sustainable development goals to market their positive contribution to some SDGs while ignoring their negative impact on others. This potential to counter unfair communication practices in the field of sustainability is also functional to the implementation of Environmental, Social and Governance (ESGs) criteria. They are now part of the strategic decision-making processes of companies and contribute to an increase in the level of transparency and accountability required by both the market and stakeholders. Therefore, the increasing adoption of SDGs and ESGs criteria in reporting processes (de Silva Lokuwaduge et al. 2020), together with greater detail regarding risks, including nonfinancial risks, will provide stakeholders with a comprehensive and transparent overview of the business and highlight its attractiveness.

### Recommendations for Further Research

This research also has several limitations, thus offering some potential areas for future research. Although other classifications of risk are possible and some risk categories have not been collected, this study explored theoretically the relationships between the sources of risks and the potential impacts they may have on business operations, thus providing the basis for future empirical testing. Achieving a holistic model of risk management requires a new interpretation that considers both the incorporation of new elements and the interaction of these elements. For this reason, the study of the effect that some types of risk have on others is proposed as a future line of research. To illustrate this, the uncertainty and long-term negative effects of epidemic outbreaks make them special cases of supply chain risks. This growing uncertainty makes it necessary to adequately monitor the new challenges that supply chain networks must face (Karmaker et al. 2020). In the same line, cyber risk can condition the effects of other risks, such as those related to the supply chain or pandemics, given the importance of information systems and communication infrastructures for management.

Since an exploratory case study approach has been used, results from this study are not suitable for generalization so a first step to validate the constructs and relations identified could be the use of multiple cases. Those cases would also allow additional weighting criteria to enhance the proposed model to focus attention either on one aspect of sustainability or on a specific level of analysis. In addition, they would be useful in identifying appropriate measures of the suggested variables and would offer better understanding of the relationships between the different risks. Apart from that, the proposed model could be completed with other interesting risks such as those derived from severe disasters. This source of risk, although better known, is still of interest for the creation of a more comprehensive and holistic model of risk management for companies, particularly due to the magnitude it takes on in a globalized world.

This research provides a conceptual model of risk as an initial step to theorize about this concept. However, the development of a guide to control organizational behavior in risk management should consider more than just the typologies and associated tools. Further research must offer new risk response matrices to draw individual and collective mitigation activities for specific types of risks (Dellermann et al. 2017). These must be linked through the development of capacities and routines, which would allow moving from the establishment of definitions and tools to the development of processes.

Finally, the current problem of finding the balance between economic growth and sustainable development makes it necessary to progress on associated risk. Future research should consider risk associated with new concepts, such as the development of green technology, the application process of which is associated with great uncertainty and risk (Sun et al. 2020).

Key Questions

1. How does risk management influence making decision process under uncertain conditions?
2. How do risk management systems relate to sustainability management systems?
